# Singular Case of Empyema

**Published:** 1844-10-05

**Authors:** 


### SINGULAR CASE OF EMPYEMA.

M. Krause, of Dantzic, describes the case of a boy, eight years of age, who was much emaciated, and presented some symptoms of phthisis, the suspicion of which, however, was abated, by finding no difference either in the circumference or movements of the chest. He had a swelling under Poupart's ligament, which presented the appearance of a lumbar abscess, was opened, and a pound of good pus evacuated. The boy died in the course of a few days, and when the body was opened, the case was discovered to be one of empyema on the left side, which communicated by a narrow sinus, through an opening in the diaphragm, along the left lumbar vertebræ, with the abscess in the groin. The lung was tuberculous, and contained a large vomica.

